# Inter-Regional Center for Automated Insulin in Diabetes (CIRDIA) and Hospital-Based Approaches to Closed-Loop Therapy in Type 1 Diabetes: Cost-Effectiveness Analysis

**DOI:** 10.2196/86690

**Published:** 2026-01-29

**Authors:** Mercia Napame, Sylvie Picard, Tony Foglia, Anne Rubenstrunk, Florence Baudoux, Francoise Giroud, Sandrine Lablanche, Sophie Borot

**Affiliations:** 1 Santelys, Loos, France; 2 CIRDIA, Point Medical, Rond-Point de la Nation, Dijon, 21000, France, 33 608232200; 3 URPS-ML-BFC, Burgundy-Europe University, Dijon, France; 4 CDOM21, CIRDIA, Dijon, France; 5 Centre Hospitalier Universitaire de Grenoble, Grenoble, France; 6 Centre Hospitalier Universitaire de Besançon, Besançon, France

**Keywords:** closed-loop, cost-effectiveness analysis, follow-up studies, hospital, treatment outcome, type 1 diabetes

## Abstract

**Background:**

Closed-loop insulin delivery is the new standard of care for patients with type 1 diabetes (T1D). However, in France, its implementation remains predominantly hospital based. Expanding access to this treatment through alternative care models looks essential.

**Objective:**

This study (cost-effectiveness analysis) compares 2 care models for people with T1D implementing a closed-loop system in France: outpatient care in the Inter-Regional Center for Automated Insulin in Diabetes (CIRDIA) and inpatient care.

**Methods:**

We conducted a cost-effectiveness analysis using retrospective observational data from individuals with T1D aged 16 years and older from the implementation of the closed loop to a 12-month follow-up either in the CIRDIA (CIRDIA group) or in a hospital center setting (hospital center [HC] group). The cost analyses were based on patient records and public databases: the French Medical Information Systems Program and the French General Nomenclature of Professional Acts. Closed-loop efficacy was assessed using a time in range (TIR) of 70 to 180 mg/dL, and closed-loop safety was assessed using the glycemia risk index (GRI), a single indicator that represents the risk of hypoglycemia or hyperglycemia and ranges from 0 (minimal risk) to 100 (maximal risk).

**Results:**

A total of 201 patients were included: 128 in the CIRDIA group and 73 in the HC group. The mean (SD) age was 43 (14) years and 46 (15) years, respectively. Mean (SD) baseline TIR was 52.9% (16%) in the CIRDIA group versus 65.9% (15.1%) in the HC group (*P*<.001), whereas mean (SD) baseline GRI was 56.4 (21) in the CIRDIA group versus 37.8 (19.8) in the HC group (*P*<.001). After 12 months, both groups achieved similar efficacy and safety outcomes with a mean (SD) TIR at 72.7% (11.6%) in the CIRDIA group versus 71.9% (10.5%) in the HC group, and a mean GRI at 30.1 (14.1) versus 30.3 (13), respectively. There were no significant between-group differences (*P*=.60 for TIR; *P*=.91 for GRI). However, the CIRDIA was associated with significantly lower management costs with a mean cost of €8373.12 (SD €427.30; €1=US $1.10 at the time of the study) per patient in the CIRDIA group versus €8814.32 (SD €192) per patient in the HC group (*P*<.001). The estimated saving was €626 per percentage point of increase in TIR and €2011 per point of reduction in GRI, indicating that the HC closed-loop initiation was dominated by the CIRDIA. The CIRDIA was less costly than HC in 8600 (86%) out of 10,000 simulations in a probabilistic sensitivity analysis.

**Conclusions:**

These findings suggest the potential of the CIRDIA to represent a viable alternative organizational model for closed-loop initiation in France, achieving comparable effectiveness at lower cost in our population. Further research with longer follow-up is warranted. From a policy perspective, the resources saved could be at least partly reallocated to support out-of-hospital closed-loop initiation centers.

## Introduction

### Background

Diabetes is a chronic disease characterized by persistent hyperglycemia, resulting from either a relative or absolute deficiency in insulin secretion or an impairment in its action. It represents a major public health challenge because of its increasing prevalence, its impact on patients’ quality of life, and the substantial economic burden on health care systems [1]. As of 2024, 588.8 million adults (aged 20‐79) worldwide were living with diabetes, a number projected to increase to approximately 853 million by 2050 [2]. In France, more than 4.5 million people are living with diabetes [3]. Among the different forms of diabetes, type 1 diabetes (T1D) is an autoimmune disease that is often diagnosed in children, adolescents, or young adults. Overall, about 7.4 million people are living worldwide with T1D, and in France, T1D accounts for approximately 320,000 individuals [3]. The management of T1D requires lifelong insulin therapy, frequent or nowadays continuous glucose monitoring (CGM), and structured patient therapeutic education [4]. Over the past decades, technological advances have progressively transformed diabetes care from multiple daily injections to external insulin pumps and subsequently to CGM, enabling the real-time tracking of glycemia [5]. These innovations have paved the way for the development of closed-loop (CL) systems, which integrate a glucose sensor, an insulin pump, and an adaptive control algorithm [5].

### Prior Work

While numerous studies have established the clinical benefits of CL systems on glycemic outcomes, evidence on the models of care for their initiation and follow-up remains limited [6-10]. The recent reimbursement of CL systems in France, and the relative novelty of studying organizational rather than purely clinical outcomes, may explain this evidence gap [11].

In France, approximately 2 years after the first reimbursement, only about 15,000 eligible patients had received CL systems—roughly a 5% coverage—despite the benefits for glycemic control [12]. This low rate is partly attributable to the centralization of CL initiation in hospital-based clinics, where waiting times are often long [13].

The Inter-Regional Center for Automated Insulin in Diabetes (CIRDIA) was developed in 2023 mainly to improve access to CL among persons with T1D. The CIRDIA is a multisite CL initiation center regrouping highly trained diabetologists, mostly in private practice. The CIRDIA—like hospital-based CL initiation centers—is based on the guidelines of the French-Speaking Diabetes Society (SFD) [4]. However, as this is a new concept of care in France, its cost-effectiveness had to be evaluated and compared to usual hospital-based care.

### Study Objectives

Evidence on the cost-effectiveness of alternative organizational models of CL initiation, such as out-of-hospital–based pathways, remains scarce. This raises the question of whether initiating CL systems in out-of-hospital settings, such as the CIRDIA, could represent a cost-effective alternative to hospital center (HC)–based initiation.

This study aimed to estimate the 1-year cost-effectiveness of CIRDIA-based CL initiation compared to HC-based initiation among patients with T1D in France from a French National Health Insurance perspective. We hypothesized that out-of-hospital–based initiation could achieve comparable effectiveness and safety while reducing costs. Evaluating this organizational model could determine whether or not the CIRDIA represents a viable alternative for the French health care system and provide the data that may be transferable to other health care systems worldwide.

## Methods

### Study Design

This is a cost-effectiveness analysis based on retrospective observational data collected between 2023 and 2024 with a 12-month follow-up as part of the routine monitoring of patients with T1D initiating CL in France. We compared 2 modes of health care delivery: the CIRDIA setting and the HC setting. The cost-effectiveness analysis compared the net monetary costs of health care intervention with a measure of its clinical effectiveness.

Accordingly, the evaluation was conducted from the perspective of the French National Health Insurance (Assurance Maladie), considering all costs covered by the payer, with a 1-year time horizon. No modeling was conducted, as all analyses relied on real-world data extracted from patient records (follow-up consultations) and public databases: the Agency for Information on Hospital Care and the French Health Insurance [14].

### Recruitment

The study included persons living with T1D, 16 years of age or older, starting for the first time a CL system. Patients with missing continuous glucose monitoring data were excluded. Participants were allocated to 1 of the 2 groups based on their care pathway: those managed directly by the CIRDIA center (CIRDIA group) and those initiated and followed by the hospital center outpatient clinic (HC group). The 2 models of care were mutually exclusive and could not be used simultaneously.

Participants from the CIRDIA group were consecutive patients who started CL between May 2, 2023, and March 30, 2024, and had at least a 12-month follow-up. Devices (insulin pump, infusion sets, insulin reservoirs, and glucose sensors) were provided by different home health care providers, as it is the rule in France. Registered nurses specialized in diabetes care and working for home health care providers are usually responsible for the technical education of the patient and connectivity issues. Participants in the HC group had CL initiated in 2023 or 2024 in 1 of the 5 HCs located in the north of France (“Haut-de-France” region) and were the patients for whom devices and technical education were provided by Santelys, a nonprofit organization acting as a home health care provider.

### Ethical Considerations

This study used retrospective observational data collected as part of the routine monitoring of persons with T1D managed on CL therapy. No additional intervention occurred beyond usual care. All data were fully anonymized before analysis in accordance with the General Data Protection Regulation. No patient could be identified directly or indirectly [15]. In line with current regulations regarding research not involving human persons, no specific ethics committee approval was required [16].

All participants had received oral and written information at the time of CL initiation about the potential use of their anonymized clinical data for research purposes. Written consent or non-opposition was obtained in accordance with French data protection and ethical regulations. This study complied with the principles of the Declaration of Helsinki and relevant national guidelines regulating the secondary use of health data.

### Interventions

The CIRDIA is a new concept in France of a multisite health care model that performs CL initiation most often during a long (about 1 h) office visit or occasionally during a day hospitalization (DH) outside of university hospitals. Its activity complies with the position statement issued by the SFD and the French National Health Authority (HAS) [17]. The main objective of the CIRDIA is to expand access to care for people living with T1D while reducing the burden on HC. Furthermore, initiating CL systems in the out-of-hospital sector is considered a strategic lever to support the sustainability of out-of-hospital diabetes care. Nevertheless, since CL initiation is predominantly performed in hospital settings, hospital-based care is considered the reference strategy. The out-of-hospital sector initiation remains underdeveloped and must demonstrate its effectiveness.

In the CIRDIA arm, CL initiation was usually followed by 3 teleconsultations and 3 consultations over 1 year. For some patients (those initiated after January 1, 2024), an additional 3-month telemonitoring period could be implemented. In the HC arm, CL initiation was carried out during DH, followed by 3 teleconsultations and 3 follow-up visits, coupled with 3 months of telemonitoring for patients initiated after January 1, 2024. In both settings, CGM data were available for the diabetologist (or the diabetes care team) to optimize patient adherence to the device [18].

### Efficacy and Safety Inputs

Because CL initiation and the 1-year time horizon did not affect mortality or lifespan, we selected an alternative measure for effectiveness. However, due to incomplete data on comorbidities and complications in 1 of the 2 study arms (HC), adverse events could not be included in the analysis. Instead, effectiveness was assessed by improvement in the time in range (TIR) 70‐180 mg/dL, while safety was assessed through a reduction in the glycemia risk index (GRI). The GRI is a composite metric that reflects both hypoglycemia and hyperglycemia risks by integrating the time spent below range (<54 mg/dL and 54‐69 mg/dL) and the time spent above range (181‐250 mg/dL and >250 mg/dL). Notably, although hemoglobin A_1c_ is frequently used as an efficacy outcome in similar studies, it is no longer systematically measured during routine consultations [19].

### Cost Inputs

We conducted the economic evaluation from a health care payer perspective, including all direct medical and nonmedical expenses reimbursed by the French National Health Insurance, expressed in euros for the year 2024. Costs were estimated using a bottom-up micro-costing approach, which is considered the gold standard in health technology cost assessment according to HAS recommendations. Because T1D belongs to the list of fully covered diseases by the French National Health Insurance, no out-of-pocket expense was considered. Moreover, because the time horizon was limited to 1 year, no discount rate was applied. Cost components were identified and calculated in line with the HAS and SFD recommendations [20,21].

Outpatient procedures and consultations were valued according to the prices from the General Classification of Professional Acts and the Common Classification of Medical Acts. Biological analyses were valued according to the Common Nomenclature of Medical Biology Acts. In addition, CL-related costs were valued in accordance with the List of Products and Services of the French National Health Insurance. The cost of DH was calculated using the Homogeneous Group of Patients with the principal diagnosis code Z451 (“Adjustment and maintenance of an infusion pump”), associated with the Hospital Stay Tariff 1794, based on prices provided by the Agency for Information on Hospital Care [22-26].

### Incremental Cost-Effectiveness Ratio

The results of a cost-effectiveness analysis were expressed in terms of incremental cost-effectiveness ratios (ICERs) and were calculated as the ratio of incremental costs to incremental health outcomes between the 2 groups. Specifically, ICERs were expressed as the additional cost per percentage point of increase in TIR and per unit of reduction in the GRI. In line with International Society for Pharmacoeconomics and Outcomes Research recommendations, negative ICERs were interpreted as situations of dominance or dominated strategies rather than reported as such. A strategy was considered to be dominated if it was more costly and less effective or more costly and equally effective. We designed, conducted, and reported this evaluation in accordance with the CHEERS (Consolidated Health Economic Evaluation Reporting Standards) guidelines [27].

### Sensitivity Analysis

As this study was based on real-world observational data rather than modeled parameters, some uncertainty may still arise from the data, potentially leading to biased estimates. According to the International Society for Pharmacoeconomics and Outcomes Research [28], deterministic sensitivity analysis was not applicable in this context. Instead, robustness was explored through subgroup analyses and through a probabilistic sensitivity analysis (PSA) to test whether the conclusions of the base-case analysis held under parameter uncertainty. A PSA was performed using 10,000 Monte Carlo simulations in which all parameters were varied simultaneously. Parameter values were sampled from predefined probability distributions: truncated normal for efficacy and safety outcomes (bounded between 0 and 100) and gamma for costs [28].

### Statistical Analysis

Data were collected using Excel (2016, Microsoft Inc.), and statistical analyses were performed with the R software version 4.4.2 (2024). Means and SDs were calculated for quantitative variables. To verify comparability between the groups, we conducted a Shapiro-Wilk test to check the normality of our variables. For normally distributed variables, we used a 2-tailed Student *t* test, and for non-normally distributed variables, the Wilcoxon signed-rank test was used. The threshold for statistical significance was set at *P*<.05.

## Results

### Overview

Overall, 201 patients aged 16 to 80 years were included in this study, including 128 CL initiations by the CIRDIA and 73 by HC. Baseline characteristics of the 2 groups are shown in Table 1. The mean age of patients initiated at CL by the CIRDIA was 43 (SD 14) years and 46 (SD 15) years for the HC arm. The gender distribution was 52% (n=66 and n=38) women and 48% (n= 62 and n=35) men in both arms, and the average BMI was 27.5 (SD 4.9) and 27.2 (SD 5.2) kg/m^2^, respectively. In the CIRDIA arm, only 17 (13%) CL initiations were performed during DH, while the remaining initiations were conducted during 1-hour office visits.

**Table 1.** Baseline characteristics of the patients included in the study by group (CIRDIA^a^ vs HC^b^).

Parameters	CIRDIA (n=128)	HC (n=73)	*P* value *(t* test/Wilcoxon test)
BMI (kg/m^2^), mean (SD)	27.5 (4.9)	27.2 (5.2)	.71
Weight (kg), mean (SD)	78.5 (14.8)	79.8 (15.3)	.55
Height (cm), mean (SD)	169 (7.9)	171 (9.3)	.048
Gender, n (%)			
Men	62 (48)	35 (48)	.95
Women	66 (52)	38 (52)	—^c^
Age class (y), n (%)			
<25	13 (10.2)	7 (11)	—
25-45	60 (46.9)	27 (37)	—
45-65	46 (35.9)	31 (42.5)	—
>65	9 (7)	7 (9.6)	—
Age (y), mean (SD)			
At pump initiation	34 (15)	45 (15)	<.001
At closed-loop initiation	43 (14)	46 (15)	.15
Pump model, n (%)			
Medtronic 780G (with Guardian 4 sensor)	99 (77)	67 (92)	—
“Control IQ” (Tandem Slim 2X pump, Dexcom G6 sensor)	17 (9)	6 (8)	—
“CamAPS” (Ypsopump, Dexcom G6 sensor)	12 (13)	—	—
Baseline glucose control, mean (SD)			
TIR^d^ (%)	52.9 (16)	65.9 (15.1)	<.001^e^
GRI^f^	56.4 (21)	37.8 (19.8)	<.001^e^

a CIRDIA: Inter-Regional Center for Automated Insulin in Diabetes.

b HC: hospital center.

c Not applicable.

d TIR: time in range 70-180 mg/dL.

e Wilcoxon test values.

f GRI: glycemia risk index.

### Efficacy and Safety Outcomes

**Figure 1.**
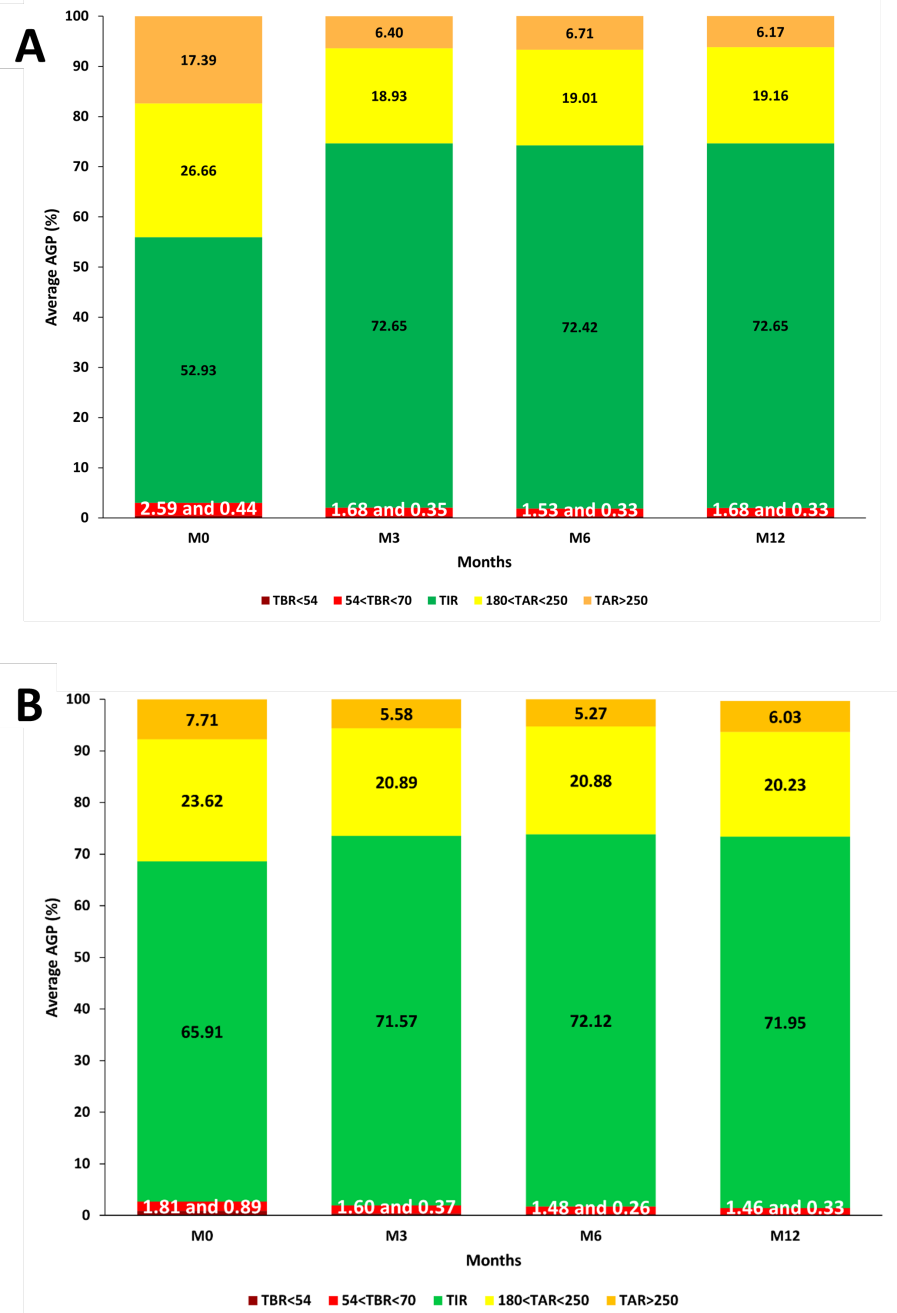
Ambulatory glucose profile for both comparison arms (A: CIRDIA group, B: hospital centers group) at baseline (M0) and after 3 months (M3), 6 months (M6), and 12 months (M12) of closed-loop use. AGP: ambulatory glucose profile; CIRDIA: Inter-Regional Center for Automated Insulin in Diabetes; TAR: time above range; TBR: time below range; TIR: time in range.

Figure 1 illustrates the changes in the ambulatory glucose profile from baseline to 1 year after initiation.

**Figure 2.**
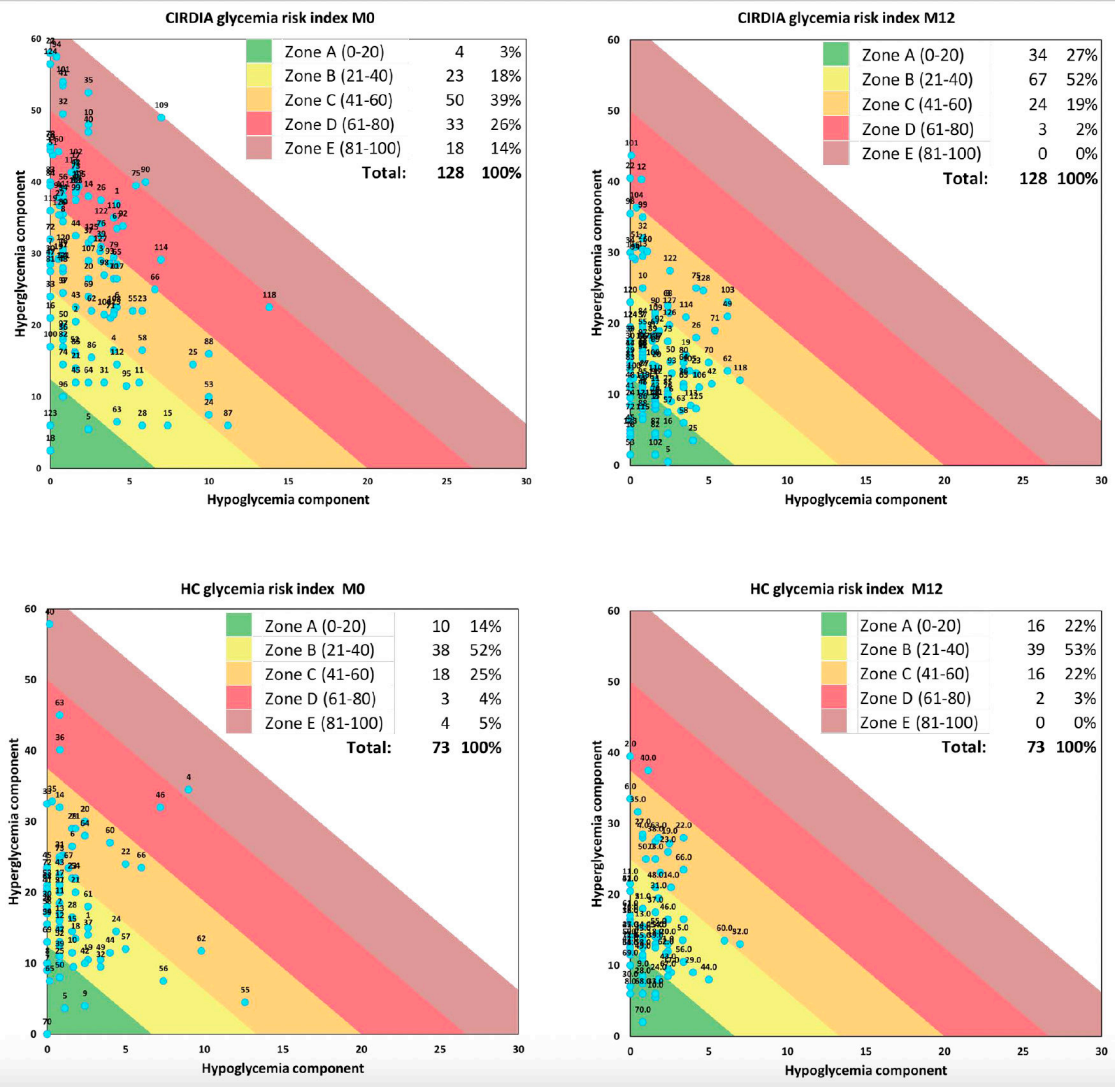
Glycemia risk index (GRI) grids at baseline (M0) and 1 year after closed-loop initiation (M12). Upper grids: Inter-Regional Center for Automated Insulin in Diabetes (CIRDIA) group; lower grids: hospital center (HC) group. Each participant is identified by a blue circle and their identification number.

Figure 2 presents GRI grids showing glycemic risk zones over the same period (zone A: minimal hypo- or hyperglycemia risk; zone E: maximal hypo- or hyperglycemia risk). At baseline, 79% (101/128) of the patients from the CIRDIA group were in the intermediate risk (zone C) or high-risk zones (zones D and E). After 1 year on CL, only 21% (27/128) remained in these GRI zones. In the HC arm, 34% (25/73) of the patients were in zones C, D, and E at baseline, and 25% (18/73) remained in these zones after 1 year.

Table 2 summarizes the overall effectiveness and safety results for the total population and according to age at inclusion. After 12 months, the mean (SD) TIR increased by 19.8 points in the CIRDIA group (from 52.9% [16] to 72.7% [11.6]) and by 6 points in the HC group (from 65.9% [15.1] to 71.9% [10.5]). Although baseline differences were significant (*P*<.001), no significant difference between groups was observed at 12 months (*P*=.60). The GRI decreased in both groups, by 26.3 points in the CIRDIA group, from 56.4 (21) to 30.1 (14.1), and by 7.5 points in the HC arm, from 37.8 (19.8) to 30.3 (13). No significant difference between groups was observed at 12 months (*P*=.91).

**Table 2.** Glycemic outcomes at baseline (M0) and 12 months (M12) after closed-loop initiation for the total population and according to age class at inclusion^a^.

Parameters		CIRDIA^b^ (n=128), mean (SD)	HC^c^ (n=73), mean (SD)	*P* value
M0^d^				
TIR^e^				
Total		52.9 (16)	65.9 (15.1)	<.001
Age class (y)				
<25		50.4 (11.4)	67.8 (14.7)	.01
25-45		48.4 (15.6)	64.7 (16.7)	<.001
45-65		57.6 (16.3)	65.8 (14)	.02
≥65		62.9 (14.2)	68.9 (14.2)	.30
GRI^f^				
Total		56.4 (21)	37.8 (19.8)	<.001
Age class (y)				
<25		59.6 (16.9)	35.3 (17.9)	.007
25-45		63 (20.7)	40.2 (22.1)	<.001
45-65		50.2 (20.1)	36.6 (17.7)	.002
≥65		39.3 (16.6)	36.7 (24.3)	.70
M12^g^				
TIR				
Total		72.7 (11.6)	71.9 (10.5)	.60
Age class (y)				
<25		70 (11.8)	77.9 (11.8)	.20
25-45		69.1 (11.7)	72 (7.9)	.20
45-65		76.7 (10.6)	72.2 (10.8)	.09
≥65		79 (6.2)	63.6 (14)	.03
GRI				
Total		30.1 (14.1)	30.3 (13)	.91
Age class (y)				
<25		33.6 (14.5)	23.5 (15.1)	.10
25-45		34.4 (13.9)	29.7 (10.7)	.12
45-65		25.3 (13.1)	30.2 (13)	.13
≥65		21.2 (7.4)	40.9 (15.3)	.01

a Values were compared using the Wilcoxon rank sum test.

b CIRDIA: Inter-Regional Center for Automated Insulin in Diabetes.

c HC: hospital center.

d M0: closed-loop initiation.

e TIR: time in range 70‐180 mg/dL.

f GRI: glycemia risk index.

g M12: 12 months after closed-loop initiation.

Subgroup analyses revealed no statistically significant differences between the CIRDIA and HC at M12, except among patients older than 65 years, for whom CIRDIA participants had higher TIR and lower GRI values (*P*=.03 and *P*=.01, respectively).

### Costs Outcomes

**Figure 3.**
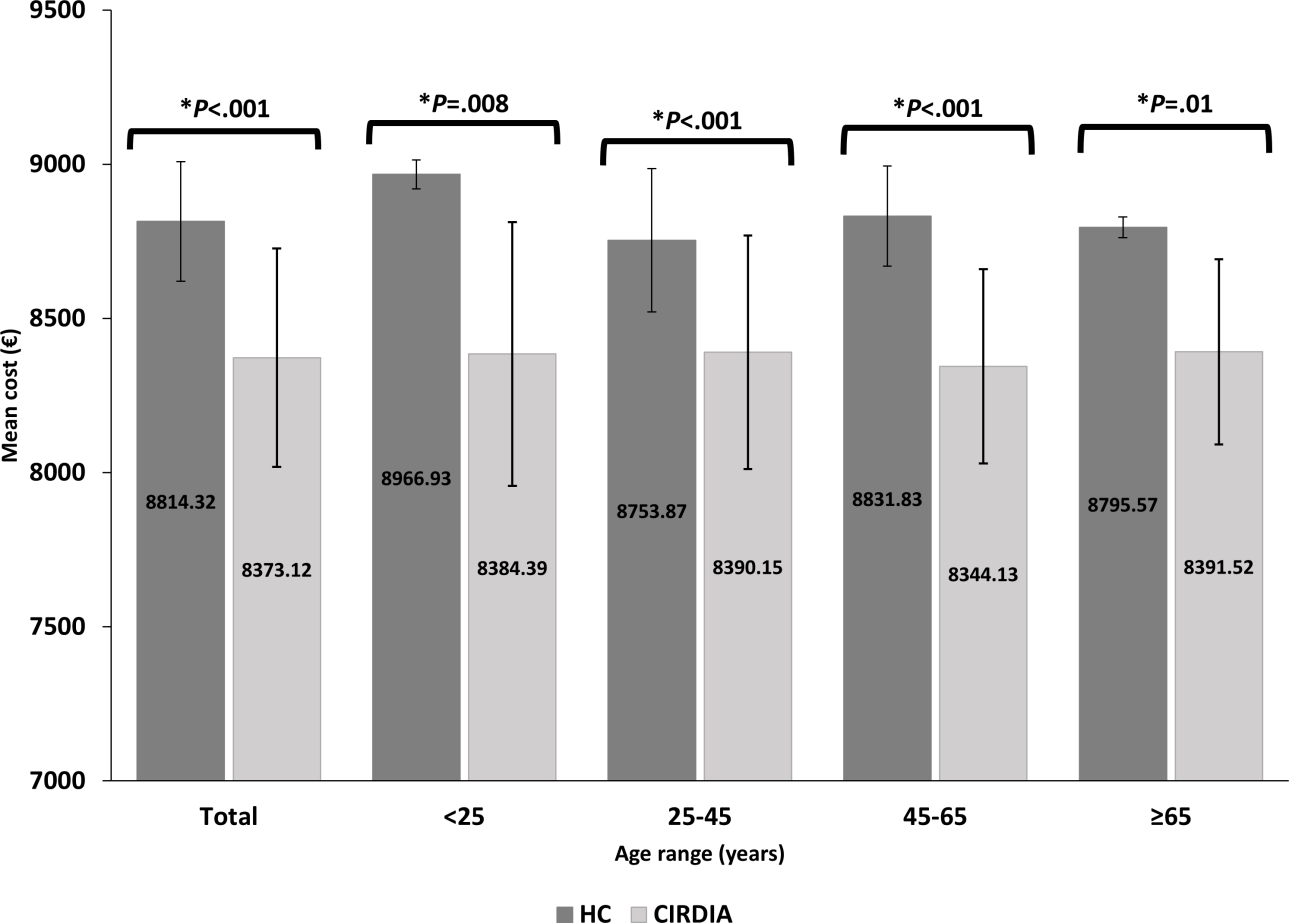
Average costs per patient according to the care setting. €1=US $1.10 at the time of the study. CIRDIA: Inter-Regional Center for Automated Insulin in Diabetes; HC: hospital centers.

We combined all cost items by type of procedure, year, and data source. Costs are expressed in euros from the French National Health Insurance perspective, and Figure 3 shows the mean costs for both comparison arms and by subgroup.

The total cost of CL insulin therapy management was €1,077,231 (1 €=1.10 US $ at the time of the study) for 128 patients initiated in the CIRDIA, which was a mean cost of €8373.12 (SD 427.3) per patient. In the HC group, the total cost was €645,991 for 73 patients, which was a mean cost of €8814.32 (SD 192) per patient. Out-of-hospital–based management was associated with significantly lower costs (*P*<.001). All cost components are shown in Table S1 in Multimedia Appendix 1.

### Incremental Cost-Effectiveness Ratio

The base-case analysis, using mean parameter values, indicated that the CIRDIA was less costly while achieving comparable effectiveness and safety to HC. This situation corresponds to dominance, with an estimated saving of €626 per additional percentage point of TIR and €2011 per point reduction in GRI, indicating that CL initiation in HC is dominated by the CIRDIA. The detailed results are presented in Table 3.

**Table 3.** Base-case cost-effectiveness and cost-safety results.

Parameters	CIRDIA^a^	HC^b^
Costs per patient (€^c^)	8373.12	8814.32
Incremental costs (€)	−441.20	N/A^d^
Mean efficacy (TIR^e^)	72.65	71.95
Incremental efficacy (increase in TIR)	0.70	N/A
Mean safety (reduction in GRI^f^)	30.11	30.33
Incremental safety	−0.22	N/A
ICER^g^	−625.83	N/A
ICSR^h^	2011.02	N/A

a CIRDIA: Inter-Regional Center for Automated Insulin in Diabetes.

b HC: hospital center.

c €1=US $1.10 at the time of the study.

d N/A: not applicable.

e TRI: time in range.

f GRI: glycemia risk index.

g ICER: incremental cost-effectiveness ratio (based on the increase of time in range).

h ICSR: incremental cost-safety ratio (based on the decreased of glycemia risk index).

### Sensitivity Analysis

**Figure 4.**
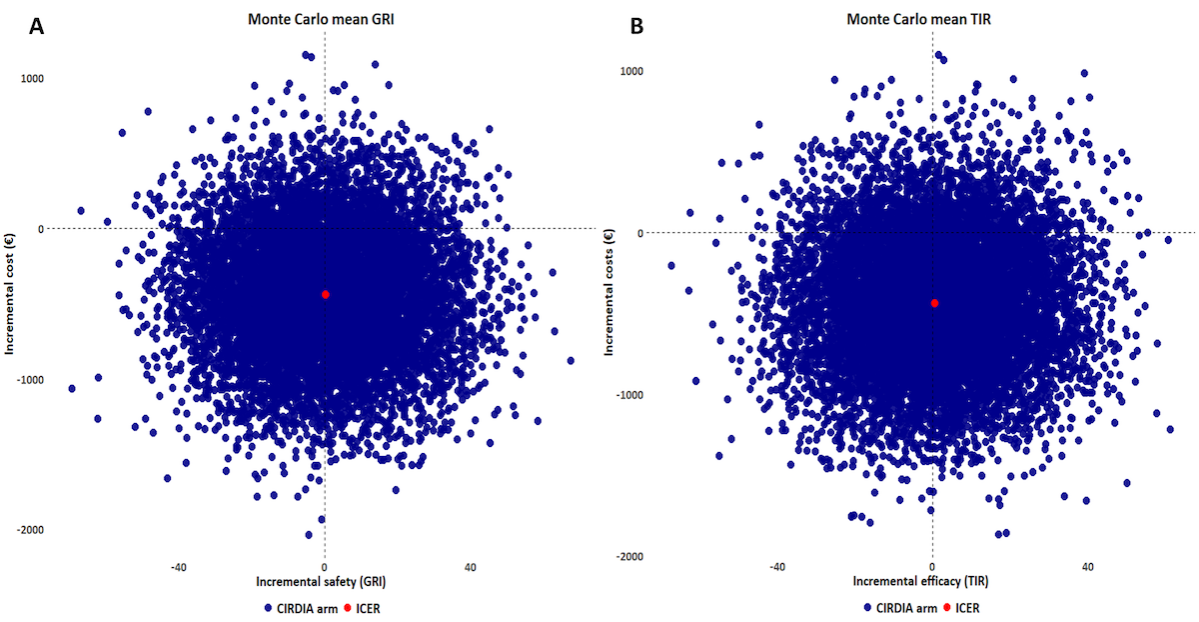
Probabilistic sensitivity analysis of 10,000 Monte Carlo simulations using glycemia risk index (GRI) (A) or time in range (TIR) (B) Inter-Regional Center for Automated Insulin in Diabetes (CIRDIA). €1=US $1.10 at the time of the study. ICER: incremental cost-effectiveness ratio.

In the PSA (10,000 simulations), the CIRDIA was less costly in 8600 (86%) of the cases. Strong dominance (less costly and more effective) was observed in 4340 (43.4%) of the simulations, while in 4270 (42.7%) of the simulations, the CIRDIA was less costly but less effective. The probability of being more or less effective was generally consistent with the base-case results. Only 1400 (14%) of the simulations placed the CIRDIA in a more costly position, being either less effective (n=700, 7.0%) or more effective (n=690, 6.9%). The scatter plot of the incremental cost-effectiveness plane is presented in Figure 4.

## Discussion

### Principal Findings

The aim of this study was to assess the cost-effectiveness of CL initiation by the CIRDIA and HC from a health care payer perspective. We examined the relationship between initiation models and the increase in TIR or reduction in GRI and whether or not an out-of-hospital CL initiation and follow-up can be achieved in a cost-effective manner compared to the usual hospital management. In our cohort, CL initiation through the CIRDIA was associated with comparable TIR and GRI values at 12 months compared to HC initiation (*P*=.60 and *P*=.91, respectively), while being consistently less costly (*P*<.001), although baseline TIR was lower and baseline GRI was higher in the CIRDIA group. Sensitivity analyses further supported these results, confirming that the CIRDIA generally remained less costly than HC across a wide range of parameter variations.

### Prior Work

To our knowledge, this is the first cost-effectiveness analysis comparing hospital-based and out-of-hospital CL initiation. However, our findings are consistent with previous studies, such as Böhme et al [29], which reported no significant differences in effectiveness between outpatient care and hospital settings in therapeutic education programs for patients with type 2 diabetes in France. Similarly, Cavassini et al [30] reported that the outpatient management of gestational diabetes was more cost-beneficial than hospital-based care in Brazil, underlining the potential economic advantages of ambulatory strategies. In the United Kingdom, Pulleyblank et al [31] also found that treatment setting had a significant impact on costs in patients with type 2 diabetes, with outpatient follow-up being less resource-intensive than hospital-based management.

Moreover, recent studies have shown that transitioning to CL reduces the GRI at 1 year [32-34], which is consistent with the trend observed in our cohort.

### Strength

One major strength of this study is the use of real-world French data, but many published economic evaluations of CL systems have so far relied mainly on modeled analyses conducted in the United States and the United Kingdom. Furthermore, the use of TIR and GRI as primary end points is relatively novel in economic evaluations, allowing for the integration of a clinically relevant weighting of risk in the assessment of glycemic control [19,35].

Finally, sensitivity analyses and subgroup explorations provided additional insights into the robustness of our results, supporting the finding that CIRDIA and HC achieved broadly comparable outcomes in terms of TIR and GRI, whereas at baseline, TIR was lower and GRI higher in the CIRDIA participants. This also underlines that prior to CL initiation, patients followed in out-of-hospital settings do not have better glucose control than those followed in hospital centers, at least in our population.

### Limitations

This study has several limitations.

First, the relatively small sample size limits the representativeness of the study population and, consequently, the robustness of the conclusions.

Second, there was an imbalance in baseline efficacy and safety outcomes between groups, which could have led to selection bias. To address this, we performed inverse probability of treatment weighting to adjust for sociodemographic characteristics as well as baseline efficacy and safety measures. After weighting, the cost advantage of the CIRDIA was maintained, and the results on effectiveness and safety suggested a potential benefit, although these should be interpreted cautiously given the limited sample size (data not shown). However, the patients who chose to start CL therapy in the CIRDIA setting might be different from the patients from the HC group in terms of prior education or other characteristics. A prospective study with better characterizations of these items will be needed.

Third, the 1-year time horizon restricts the evaluation to the short term and does not allow assessment of long-term effectiveness or costs, although this choice was justified by the specific objective of analyzing the initiation phase of CL.

Fourth, because the costs were assessed using French Health Insurance (Assurance Maladie) rates, the results may not be generalizable to other health care systems. However, this study suggests that CL initiation in an outpatient setting is feasible, safe, and probably less expensive than the inpatient setting, regardless of the health care system.

Fifth, we cannot exclude a bias in the recruitment of HC patients as it is possible that the patients sent to Santelys home health care provider by the hospital teams might have a different (here better) control compared to other HC patients. However, as patients are from 5 different hospitals, it is unlikely that this happened in all of the hospitals.

Finally, missing information on complications and comorbidities in the hospital arm may have led to an underestimation of certain costs (eg, retinopathy-related tests), although this does not appear to alter the overall trend observed.

Nevertheless, the data from the French Closed-Loop Observatory (OB2F) indicate that outpatient initiation is already widespread, reinforcing the relevance of investigating this organizational model [18].

### Conclusion

This cost-effectiveness analysis compared 2 models of CL initiation for patients with T1D: a conventional hospital-based model and an out-of-hospital–based model supported by the CIRDIA.

Although baseline TIR was lower and baseline GRI was higher in the CIRDIA out-of-hospital setting compared to the HC setting, our results showed no significant differences in efficacy or safety outcomes between the 2 approaches. However, the CIRDIA setting was associated with lower management costs. While the patients who choose to initiate a CL system in the CIRDIA setting are probably not the same as the patients who choose to initiate CL in hospitals, these real-life findings suggest that the CIRDIA may represent a viable alternative organizational model for CL initiation in France, as it combines efficacy and savings.

Future research should assess whether these results hold over longer time horizons (eg, 5 or even 10 y) and from broader perspectives, such as a societal perspective that incorporates quality of life and indirect costs. Such work would enable cost-utility analyses to complement our cost-effectiveness findings.

From a policy perspective, the resources saved through out-of-hospital CL initiation could be reallocated to organizations such as the CIRDIA, which bring together highly trained diabetologists and uphold high-quality standards. This would allow persons living with T1D to choose their CL initiation setting, ensure early access to new technologies, and benefit the overall health care system through a cost-effective model.

## Supplementary material

10.2196/86690 Multimedia Appendix 1 Costs components for closed-loop system management.

## Abbreviations

CGM: continuous glucose monitoring
CHEERS: Consolidated Health Economic Evaluation Reporting Standards
CIRDIA: Inter-Regional Center for Automated Insulin Therapy
CL: closed-loop
DH: day hospitalization
GRI: glycemia risk index
HAS: French National Health Authority
HC: hospital center
ICER: incremental cost-effectiveness ratio
PSA: probabilistic sensitivity analysis
SFD: French-Speaking Diabetes Society
T1D: type 1 diabetes
TIR: time in range
