# Supplementary material for: Inter-Regional Center for Automated Insulin in Diabetes (CIRDIA) and Hospital-Based Approaches to Closed-Loop Therapy in Type 1 Diabetes: Cost-Effectiveness Analysis
Source: JMIR Diabetes. 2026 Jan 29;11:e86690. doi: 10.2196/86690 (PMC12854399; doi:10.2196/86690)
Supplement: Multimedia Appendix 1 [file diabetes-v11-e86690-s001.docx]

**Table S1:**  Costs components for Closed-Loop System Management.

| **Components** | **Code** | **Year** | **Value (€)** | **Data source** |
| --- | --- | --- | --- | --- |
| **Outpatients** | GHS: 1794 | 2023 | 657,65 | GHS tariffs |
| **Outpatients** | GHS: 1794 | 2024 | 695.64 | GHS price |
| **Medical consultation** | CS + MPC+MCE+MCS | 2023 | 52 | NGAP |
|  | CS + MPC+MCE+MCS | 2024 | 53.5 | NGAP |
|  | CS+MCS+MCE | 2025 | 56.5 | NGAP |
| **Teleconsultation** | TC+MCE | 2023-2024 | 52 | NGAP |
|  | TC | 2025 | 30 | NGAP |
| **Tele monitoring (for patients on CL systems after 01/01/2024)** | TS | 2024 | 70 | NGAP |
| **Technical training package for closed-loop system initiation** | 1169758 (780G) | 08/2023 | 390.91 | JO + LPP |
|  | 1113261 (CIQ) | 08/2023 | 390.91 | JO + LPP |
|  | 1179260 (CAMAPS) | 17/02/2025 | 390.91 | JO + LPP |
| **Daily package: license / algorithm / sensor / transmitter** | 1111990 (780G:  Guardian sensor 4) | 07/2023 | 8.25 | JO + LPP |
|  | 1139823 (CIQ) without sensor | 08/2023 | 3.57 | JO + LPP |
|  | 1152150 (CAMAPS) without sensor | 10/2023 | 3.33 | JO + LPP |
| **Daily package: insulin pump (consumables)** | 1182470 (780G) | 08/2023 | 6.85 | JO + LPP |
|  | 1168115 (CIQ) | 08/2023 | 6.85 | JO + LPP |
|  | 1105920 (CAMAPS) | 10/2023 | 6.85 | JO + LPP |
| **Daily package: care service** | 1197789 (780G) | 08/2023 | 4.69 | JO + LPP |
|  | 1122060 (CIQ) | 08/2023 | 4.69 | JO + LPP |
|  | 1113427 (CAMAPS) | 10/2023 | 4.69 | JO + LPP |
| **Daily package: DEXCOM G6** | 1173056 | 05/2024 | 6.57 | JO + LPP |
| **Emergency package** | FU2 (patients 16 - 45 years) | 2024 | 35.13 | ATIH |
| **HbA_1c_** | 1577 | 2023 | 4.98-5.17 | NABM, Biol'AM |
|  | 1577 | 2024 | 2.29-4.91 | NABM, Biol'AM |
|  | 1577 | 2025 | 2.28-2.29 | NABM, Biol'AM |
| **Nursing procedures: blood sampling, lipid profile, creatinine clearance** | AMI: coef 1.5 | 2023-2025 | 3.15 | NGAP |
| **Lipid profile** | 996 | 2023 | 4.18-4.34 | NABM, Biol’AM |
|  | 996 | 2024 | 4.03-4.13 | NABM, Biol’AM |
| **Creatinine clearance** | 627 | 2023 | 1.58-1.64 | NABM, Biol’AM |
|  | 0592 | 2024 | 1.52-1.56 | NABM, Biol’AM |
| **Cardiologist consultation** | CSC+MCC | 2023 | 52.5 | NGAP |
| **Resting ECG (for individuals aged ≥45 years)** | DEQP001 | 2023-2024 | 14.26 | CCAM |
| **Ophthalmologist consultation** | CS+MPC+MCS | 2024 | 30 | NGAP |
| **Fundus examination** | BGQP002 | 2024 | 28.29 | CCAM |
| **TSH** | 1208 | 2023 | 5.22-5.42 | NABM, Biol’AM |
|  | 1208 | 2024 | 5.03-5.15 | NABM, Biol’AM |
